# C-Terminal Moiety of Tudor Contains Its *In Vivo* Activity in *Drosophila*

**DOI:** 10.1371/journal.pone.0014378

**Published:** 2010-12-17

**Authors:** Joël Anne

**Affiliations:** Department of Developmental Genetics, Deutsches Krebsforschungszentrum, Heidelberg, Germany; Katholieke Universiteit Leuven, Belgium

## Abstract

**Background:**

In early *Drosophila* embryos, the germ plasm is localized to the posterior pole region and is partitioned into the germline progenitors, known as pole cells. Germ plasm, or pole plasm, contains the polar granules which form during oogenesis and are required for germline development. Components of these granules are also present in the perinuclear region of the nurse cells, the nuage. One such component is Tudor (Tud) which is a large protein containing multiple Tudor domains. It was previously reported that specific Tudor domains are required for germ cell formation and Tud localization.

**Methodology/Principal Findings:**

In order to better understand the function of Tud the distribution and functional activity of fragments of Tud were analyzed. These fragments were fused to GFP and the fusion proteins were synthesized during oogenesis. Non-overlapping fragments of Tud were found to be able to localize to both the nuage and pole plasm. By introducing these fragments into a *tud* mutant background and testing their ability to rescue the *tud* phenotype, I determined that the C-terminal moiety contains the functional activity of Tud. Dividing this fragment into two parts reduces its localization in pole plasm and abolishes its activity.

**Conclusions/Significance:**

I conclude that the C-terminal moiety of Tud contains all the information necessary for its localization in the nuage and pole plasm and its pole cell-forming activity. The present results challenge published data and may help refining the functional features of Tud.

## Introduction

In a wide variety of animals, germ cells are formed in a specialized region of the egg cytoplasm, called the germ plasm, which contains characteristic electron-dense organelles, the germinal granules [1], [2]. In *Drosophila*, assembly of the germinal granules, or polar granules [3], requires the function of maternal-effect genes. Among these genes are *oskar, vasa* (*vas*), *tudor (tud)*, and *valois* (*vls*) which are essential for the formation of pole cells, the germline progenitors [4]. These genes produce proteins that localize to the polar granules [5]–[9]. Three polar granule components, Tud, Vls, and Vas, are also present in a distinct structure at the periphery of nurse cell nuclei, the nuage [6], [9], [10].

*tud* was the first member of the posterior group of genes identified in *Drosophila*. *tud* is necessary for germline specification but is largely dispensable for abdomen formation [11], [12]. Polar granules are reduced in number and size in strong *tud* mutants [11], [12]. By comparison to the other nuage and polar granule components Tud displays specific characteristics: it is not required for the repression of heterochromatin retrotransposons [13] and furthermore Tud is bound to the fibrous material connecting polar granules and mitochondria [14]. A role for Tud in the association of polar granules with mitochondria is questionable because in *tud* null mutant oocytes the polar granules are abnormal in size and electron density, but still remain associated with mitochondria [12]. However, *tud* is involved in the transport of mitochondrial ribosomal RNAs from mitochondria to polar granules [14] and thus the assembly of mitochondrial-type ribosomes in these structures, which is necessary for pole cell formation [15].

The main structural feature of Tud protein is the presence of multiple repeats of a conserved domain, called the Tudor domain, which is found in proteins from a wide variety of organisms (reviewed in [16]). Tudor domain-containing proteins have been shown to interact with other proteins and efficient binding requires either methylated arginine or methylated lysine residues in the target protein [17]–[21]. For example, the Tudor domain of the Survival Motor Neuron protein binds directly to Sm proteins during spliceosome assembly [17], [21]–[23]. Two repeats of a Tud domain were identified in the N-terminal domain of the Fragile X Mental Retardation Protein, and one of these domains was shown to interact with methylated lysine [24]. Structural analysis of Tud domains from different proteins revealed that these domains can either fold into a single barrel-like structure [23] or form an intertwined structure consisting of two Tud domains [25]. Tud protein was shown in vitro to interact with the Capsuléen methyltransferase and Vls, which are components of the methylosome in *Drosophila* [9], [26]. The methylosome is responsible for the production of symmetrical di-methyl-arginine (sDMA) residues. Tud has been shown to interact with Sm [26] and Aubergine [27], [28] proteins in an sDMA-dependent manner, confirming that Tud, like other proteins in the family, bind to methylated substrates.

Characterization of multiple *tud* alleles, as well as the analysis of transgenic lines expressing tagged-Tud versions, have been reported [29]. Embryos produced by females carrying certain *tud* alleles form some germ cells, and these embryos grow up into fertile adults [11], [29]. One such mutant includes *tud* ^A36^ which has a point mutation in the first Tudor domain, suggesting that this Tudor domain may not be crucial for germ cell formation. By contrast, *tud* ^B42^, a point mutation in another Tudor domain, produces no germ cell. Because point mutations in *tud* ^A36^ and *tud* ^B42^ affect the equivalent arginine in these Tudor domains, a more specific function in germ cell formation for the domain affected in *tud* ^B42^ has been suggested [29]. However, because Tud^B42^ is not localized at the posterior pole [29], whether the Tudor domain altered in Tud^B42^ is necessary for the biochemical activity of Tud remains to be elucidated.

One Tud version, called mini-Tud Δ1 protein, localized to the nuage but not to the germ plasm whereas another one, called mini-Tud Δ3 protein, failed to localize to the nuage during late oogenesis but localized well to the germ plasm of oocytes and early embryos. Because mini-Tud Δ3, but not mini-Tud Δ1 protein, is able to support germ cell formation, the authors conclude that Tud localization to the nuage is not absolutely required for germ cell formation and that specific Tudor domains control Tud protein localization [29].

Here I sought to determine by analyzing the activity of contiguous fragments which part of Tud mediates its localization in the nuage and pole plasm during oogenesis and to identify the functional part of Tud.

## Results

### Multiple domains in Tud direct its localization to nuage and pole plasm

**Figure 1:**
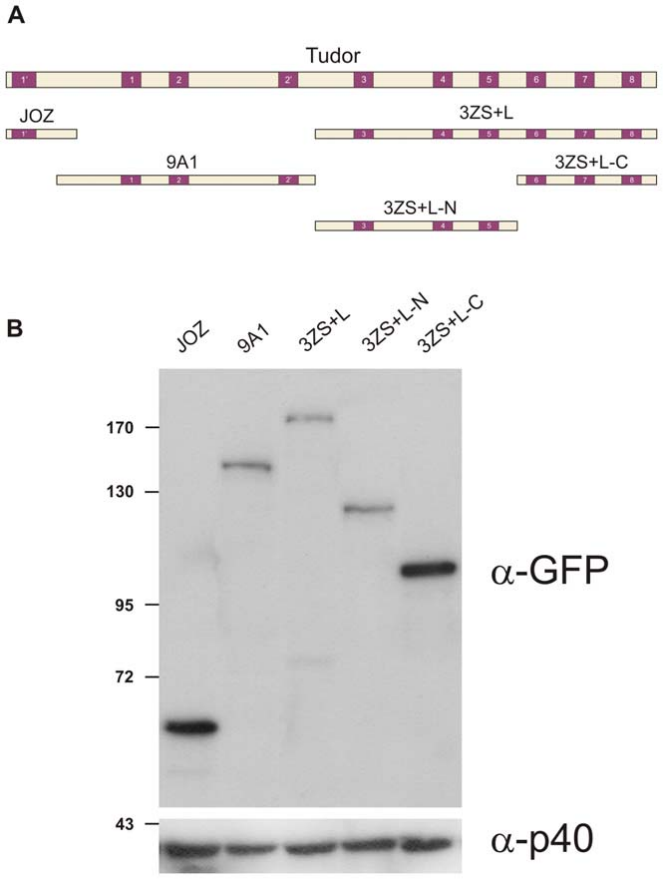
Synthesis of GFP-Tud fusion proteins in *Drosophila* ovaries. (**A**) Representation of the Tud protein with Tudor (1–8) and Tudor-like (1′-2′) domains depicted in purple. Fragments of Tud [33] used to design the transgenes are indicated below the map. (**B**) (Upper panel) Western blot analysis of GFP-Tud fusion proteins synthesized in ovaries of transgenic females using anti-GFP antibodies followed by alkaline phosphatase-conjugated antibodies. (Lower panel) The blot was then probed for ribosomal P40, as loading control.

The *tud* gene encodes a relatively large protein of 2515 amino acid residues with an approximately molecular mass of 285 kDa [6]. By using hydrophobic cluster analysis the Tud protein has been reported to contain 8 Tudor and 2 more divergent Tudor-like domains [30] (Figure 1A). Based on sequence similarity, an additional domain (located between domains 2′ and 3) has been putatively identified by Talbot et al. [31]. Several discrete segments of Tud were previously shown to bind either Vls [9] or SmB [26] and thus I was interested to find out which parts of Tud could direct its localization to nuage and pole plasm. For this purpose transgenic lines that synthesize a series of different Tud polypeptides fused to GFP were generated. The transgenes were expressed during oogenesis under the control of the *vas* promoter [32]. Three segments of Tud, JOZ (amino acid residues 3-273), 9A1 (residues 198-1199) and 3ZS+L (residues 1198-2515) [33] together comprising the complete Tud protein (Figure 1A), were cloned in frame with the GFP protein.

The relative amount of Tud polypeptides synthesized in transgenic females was first monitored. Western blot analysis of ovarian extracts using anti-GFP antibodies revealed detectable levels of Tud-GFP polypeptides in all transgenic flies (Fig. 1B).

**Figure 2:**
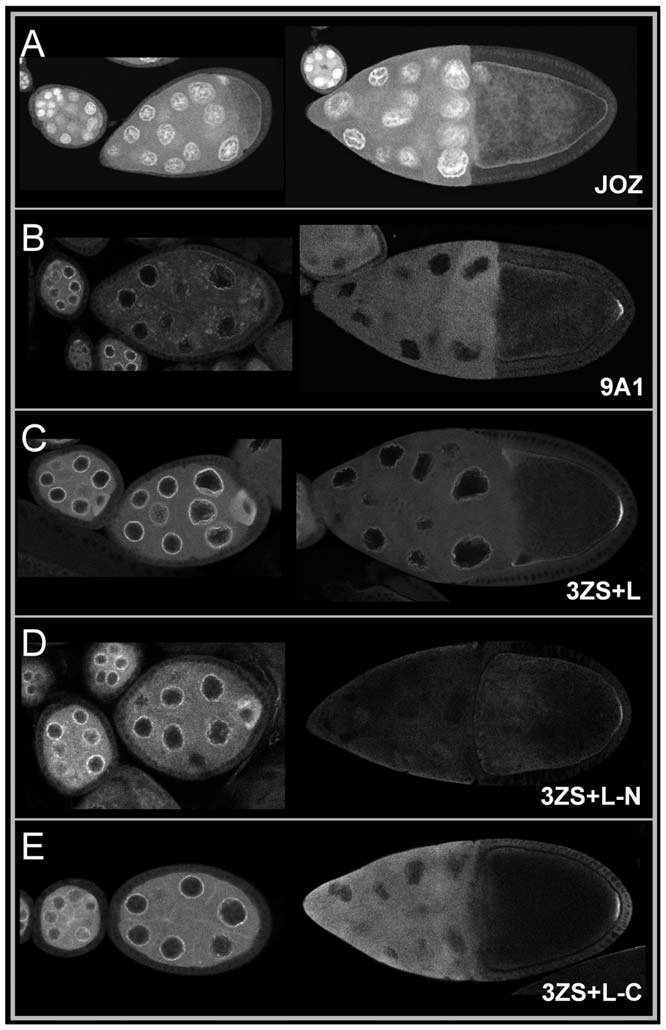
Spatial distribution of GFP-Tud fusion proteins during oogenesis. GFP-Tud proteins were detected in fixed egg chambers. Panels of the left display previtellogenic egg chambers whereas panels on the right exhibit vitellogenic egg chambers. (**A**) The GFP-Tud-JOZ fusion protein accumulated in nurse cell and oocyte nuclei. In a stage 10 egg chamber no localization of this protein could be detected at the posterior pole of the oocyte. (**B**) The GFP-Tud-9A1 fusion protein could be found in the nuage and dispersed in punctuate structures in the cytoplasm. In stage 10 egg chamber the fusion protein accumulated at the posterior pole of the oocyte. (**C**) The GFP-Tud-3ZS+L fusion protein was present in both nuage and oocyte of previtellogenic egg chambers and localized in the pole plasm of a late stage 9 egg chamber. (**D–E**). Both GFP-Tud- 3ZS+L-N and GFP-Tud-3ZS+L-C fusion proteins corresponding to the N- and C-moieties of GFP-Tud-3ZS+L, respectively, displayed a pattern of distribution in both nuage and pole plasm similar to the original GFP-Tud- 3ZS+L fusion protein with the exception that the staining intensity was lower and that the pole plasm accumulation of the N-terminal moiety was significantly reduced.

The distribution of the Tud polypeptides in the ovaries of transgenic flies was then determined. With the exception of the Tud-JOZ polypeptide, which accumulates in the nuclei of both nurse cells and oocyte (Fig. 2A), the two other Tud polypeptides were detected in the nuage and pole plasm (Fig. 2B–C). Notably, the Tud-9A1 polypeptide was also found in particles dispersed in the cytoplasm of nurse cells and in the oocyte of previtellogenic egg chambers (Fig. 2B, left panel). Tud-9A1 was found to be targeted to the posterior cortex of vitellogenic stage 10 oocytes but was undetected in the nuage at this stage (Fig. 2B, right panel). Interestingly the C-terminal 3ZS+L polypeptide was still detected in the nuage when it accumulated at the posterior pole of the oocyte (Fig. 2C, right panel). This pattern of distribution resembled that seen for the full length Tud protein. When the 3ZS+L encoding sequence was cleaved into two segments producing the 3ZS+L-N and 3ZS+L-C polypeptides (residues 1198–1981 and 1941–2515, respectively, [9] Anne and Mechler, 2005), both polypeptides could still be targeted to the oocyte posterior pole, but with a lower efficiency than the original 3ZS+L fragment (Fig. 2D–E). The ability of the different non-overlapping Tud polypeptides to localize to the nuage and pole plasm indicates a functional redundancy in Tud concerning its subcellular targeting.

**Figure 3:**
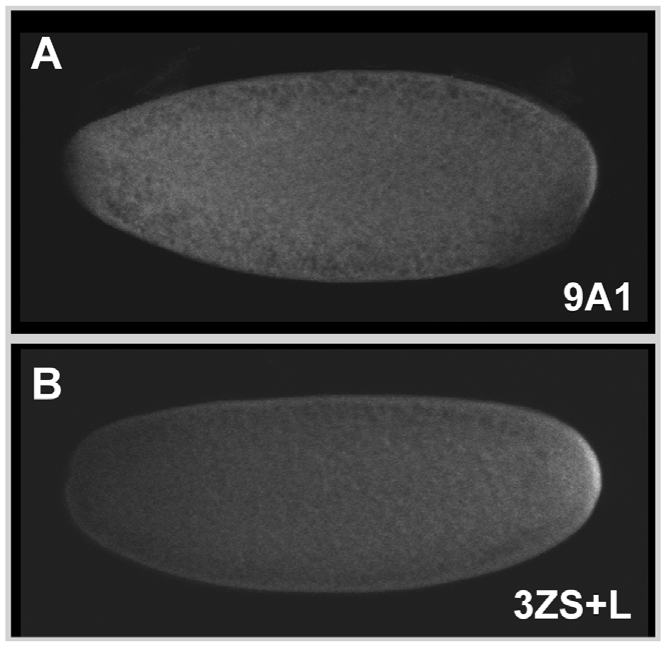
Spatial distribution of GFP-Tud fusion proteins during early embryogenesis. GFP-Tud proteins were detected in fixed 0-2 hour embryos produced by females synthesizing the GFP-Tud-9A1 (**A**) or GFP-Tud-3ZS+L (**B**) fusion proteins. Although both fusion proteins could be detected at the posterior pole of the early embryo, the pole plasm localization of GFP-Tud-9A1 was significantly reduced compared to that of GFP-Tud-3ZS+L.

Whether the localization of two GFP fusion proteins, 9A1 and 3ZS+L, is maintained at the posterior pole during early embryogenesis was then investigated. Both proteins were detected at the posterior pole of early embryos. However, the staining signal was found to be reduced for GFP-9A1 (Fig. 3A) compared to GFP-3ZS+L (Fig. 3B).

### Tud activity toward pole cell formation resides in its C-terminal moiety

**Figure 4:**
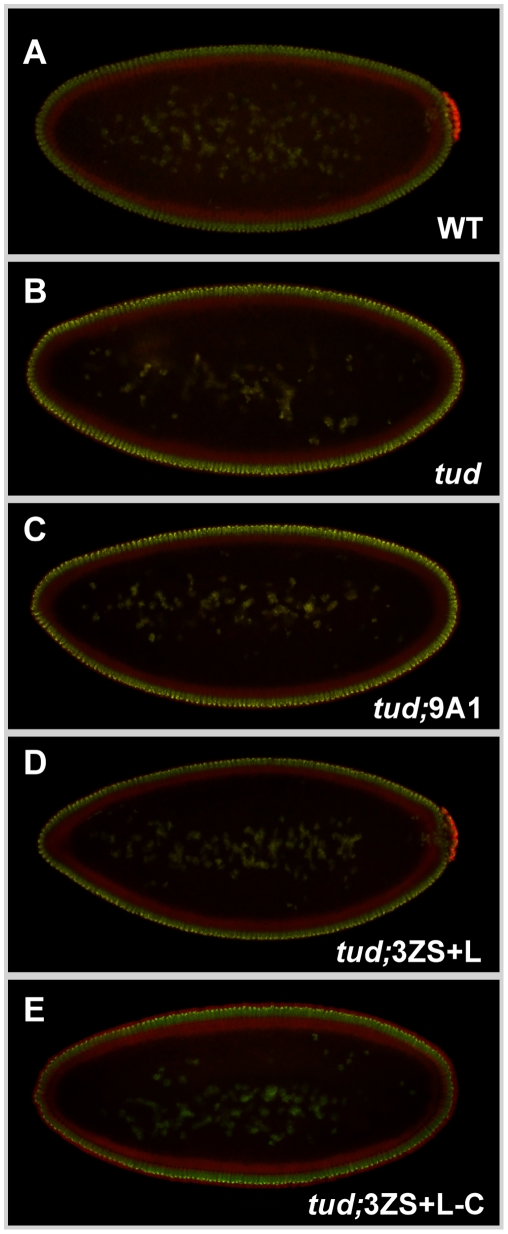
A C-terminal segment encompasses the Tud function. (**A**) Wild-type embryos at the syncytial blastoderm stage, or corresponding embryos derived from (**B**) homozygous *tud^1^* females and *tud^1^* females expressing (**C**) *GFP-Tud-9A1*, (**D**) *GFP-Tud-3ZS+L*, and (**E**) *GFP-Tud-3ZS+L-C* transgenes. Vas (red) and DNA (green). Only the *GFP-Tud-3ZS+L* transgene can restore pole cell formation in *tud^1^* embryos.

These results showing that distinct Tud segments could be incorporated in the pole plasm prompted me to investigate whether any of the Tud polypeptides displayed *tud* activity. The different GFP-Tud transgenes were introduced into a *tud^1^* background and the formation of pole cells in eggs laid by transgenic *tud^1^* females was analyzed. This mutation corresponds to a strong *tud* allele, which displays a strict grandchildless phenotype [11]. Moreover, *tud^1^* ovaries synthesize no detectable Tud protein [29]. From the five tested transgenic lines, only the largest C-terminal Tud-3ZS+L construct was found to be able to restore the formation of pole cells (Fig. 4D). All other transgenes were negative. These results indicate that the expression of *tud* sequences encoding three Tudor domains is sufficient to target the Tud polypeptides into the pole plasm but inadequate to promote pole cell formation. Synthesis of a larger *tud* fragment encompassing the C-terminal moiety of the protein is thus necessary for pole cell formation.

### Tud-3ZS+L is recruited by the short Osk protein isoform

**Figure 5:**
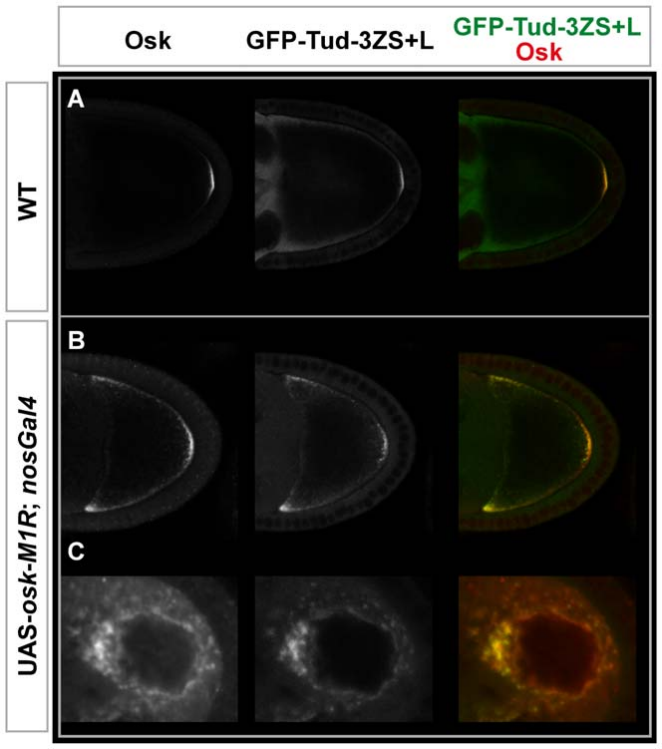
Recruitment of GFP-Tud3ZS+L by short Osk protein. Distribution of Osk and GFP-Tud-3ZS+L in (**A**) wild-type and (**B**) UAS-*osk*-M1R; *nosGal4* stage 9 egg chambers. Osk protein and GFP-Tud-3ZS+L co-localized only in pole plasm. Overexpression of the short form of Osk during oogenesis led to ectopic accumulation of Osk and GFP-Tud-3ZS+L (Upper panels) at the anterior pole of the oocyte and (Lower panels) in cytoplasmic particles in the nurse cells.

As Tud protein is present in polar granules [6] and the short Osk isoform recruits all components of the polar granules [7], whether this Osk form directs the incorporation of Tud-3ZS+L in the pole plasm was investigated. For this analysis the *UAS-osk-M1R-K10* transgene in which the 3′ UTR of the *osk* mRNA has been replaced by that of *K10* and the initiation codon of the long Osk isoform substituted by a codon encoding an arginine residue was used. This transgene, in combination with the *nos::Gal4* germline driver, directed the synthesis of high levels of the short Osk isoform in both nurse cells and oocyte [34]. Examination of *UAS-osk-M1R-K10/P_vas_-GFP-Tud-3ZS+L; nos-Gal4* ovaries showed a complete co-localization between Tud-3ZS+L and short Osk in the nurse cells and oocyte (Fig. 5B–C), indicating that short Osk was able to recruit Tud-3ZS+L in the pole plasm.

### Tud production during early embryogenesis

**Figure 6:**
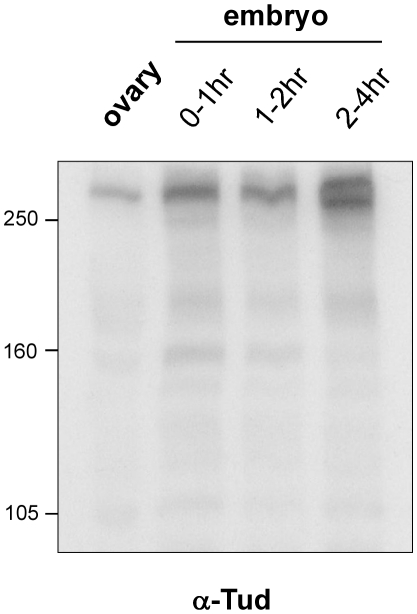
Immunoblot detection of Tud protein during oogenesis and early embryogenesis. Protein extracts from ovaries and early embryos were run on 6% SDS-polyacrylamide gels, transferred to an Immobilon-P membrane and probed with anti-Tud antibodies (TUD65). The relative molecular masses of the marker protein bands are indicated on the left.

Previous analysis of Tud production during embryogenesis revealed the occurrence, in addition to the full-length (285 kDa) protein of two additional polypeptide bands of lower molecular masses (205 and 135 kDa) in early embryos (0–2 hours of development) [6]. Primary antibodies used in this study were rabbit anti-Tud made against an internal portion of Tud (886-1199) and a second antiserum directed against the carboxy-terminal region of Tud; both antibodies gave the same pattern [6]. To confirm these results western blot analysis of early embryos was performed using the primary antibodies made against the C-terminal region of Tud protein (residues 2189–2515) (TUD65) [14]. This antiserum recognizes a protein of the predicted size in ovarian and embryonic extracts (Fig. 6). In contrast to the previously reported pattern, it did not recognize lower molecular mass polypeptides in early 0–2 hour embryos. In 2–4 hour embryonic extracts it does, however, recognize two high molecular mass polypeptides. From these results I thus conclude that Tud protein remains largely uncleaved before pole cells form.

## Discussion

### Tud functional region

As an initial step to elucidate the biochemical activity of Tud I asked whether a limited region of the molecule would support the formation of pole cells. The expression of a series of essentially non-overlapping fragments of Tud fused to GFP revealed that the C-terminal half of Tud containing six Tudor domains (the 3ZS+L fragment) allowed the formation of pole cells in *tud* embryos laid by transgenic females. This finding is partially in contrast to the data of Arkov et al. [29] reporting that the first Tudor-like domain should be associated with the five C-terminally located Tudor domains to produce a functional Tud protein. The reason for including the first Tudor-like domain resides in the characterization of the *tud^A36^* mutation containing a substitution in this domain [29]. However, this mutation supports a significant level of germ cell formation, as does the *tud^4^* mutation affecting the domain 4 (which corresponds to domain 7 in Arkov et al. [29]), whereas *tud^B42^* in domain 7 (which corresponds to domain 10 in Arkov et al. [29]) completely abolishes germ cell formation [29]. Whether a shorter Tud polypeptide containing less than 6 Tudor domains but more than 3, as tested here, would be able to promote pole cell formation in *tud* mutant remains to be investigated.

Additionally, an HA-tagged version of Tud containing the first and the last five Tudor domains (produced from the *mini*-*tud Δ3* construct) has been reported to localize to the pole plasm, albeit at a much reduced level compared to full-length HA-Tud, and to partially rescue *tud* mutation [29]. By contrast, the 9A1 fragment, which localizes at the posterior pole of the early embryo at a reduced level compared to the 3ZS+L fragment, does not rescue *tud* mutation. Although it is not clear whether the absence of rescuing activity of 9A1 correlates with the reduction of its posterior localization, taken altogether, these results suggest that the 9A1 fragment lacks functionnal sequences required for Tud activity. These elements are only present in the 3ZS+L fragment.

Interestingly, previous western blot analyses performed using antibodies directed against the C-terminal region of Tud revealed the occurrence of two additional polypeptide bands of lower molecular masses in early embryos [6]. The smallest band of 135 kDa may correspond approximately to the size of the 3ZS+L fragment. The present finding that the fragment containing the six C-terminal located Tudor domains is sufficient to direct pole cell formation suggests that Tud may be processed in early embryos in order to be fully active. The production of Tud proteins during early embryogenesis was checked using specific anti-Tud antibodies but in contrast to previously reported findings the present data do not support processing of Tud protein during early embryogenesis. Whether internal cleavage of Tud should be proceeded to generate Tud fragment to fulfill the Tud function remains therefore an open question. I nevertheless conclude that the functional activity of Tud resides in its C-terminal part.

### Nuage and pole plasm localization of Tud

The ability of Tud segments to localize to the nuage or the pole plasm were tested by fusing these segments to GFP and then by visualizing their distribution in early and stage 10 transgenic egg chambers. In stage 10 egg chambers the two Tud polypeptides 9A1 and 3ZS+L, which encompasse most of the Tud protein, accumulated in the pole plasm but only the C-terminal fragment could be detected in the nuage. Surprisingly an HA-tagged version of Tud containing the first 1544 amino acids and the last 72 amino acids (produced from the *mini*-*tud Δ1* construct) has been reported to localize to the nuage but not to the germ plasm [29]. Because the 9A1 fragment is contained within this construct it was surprising to detect the 9A1 fragment at the posterior pole of stage 10 oocyte. The reason for this discrepancy remains elusive. When the 3ZS+L fragment was divided into two parts both segments were visualized in the pole plasm but not in the nuage. It should be noted that despite a comparable nuage accumulation of these two fragments the targeting of the 3ZS+L-C fragment to the pole plasm is more efficient than that of the 3ZS+L-N fragment. The possibility that the 3ZS+L-N fragment becomes unstable in late egg chambers cannot be ruled out. Interestingly, truncation of the last 32 amino acids (Tud^A7^) abrogates pole plasm localization, suggesting that sequences outside of the Tudor domains are essential for correct targeting of Tud at this location. In contrast to stage 10 egg chambers all tested constructs were able to localize to the nuage during early oogenesis. Although nuage localization is progressively lost during oogenesis it is possible that the nuage-localized Tud proteins present in early egg chambers correspond to the ones targeted to the pole pasm at stage 9. Whether robust pole plasm accumulation requires nuage localization cannot therefore be confirmed or invalidated.

## Materials and Methods

### Fly strains

The recipient stock for P element transformation used in this study was *w^1118^*. The *UAS*-*osk*-M1R line [34] was kindly given and A. Ephrussi. Flies were grown at 25°C on corn/agar medium. Dry yeast was added to the medium the day before females were dissected for ovary preparation.

### Molecular Biology

Plasmid constructs were generated by amplification of the desired fragments by PCR (High Fidelity PRC Master; Roche), and were subcloned into the *P_vas_-GFP* vector [32]. The *P_vas_-GFP* vector and the *tud* cDNA plasmids were kindly provided by A. Nakimura and R. Boswell, respectively.

### Detection of GFP signal

Ovaries were dissected in PBS, fixed in 4% paraformaldehyde in PBS for 10 minutes, washed four times for 10 min in PBT, and mounted in Glycerol:PBS, 1∶1, onto glass slides. Data were acquired as single images with a Nikon Ellipse microscope.

### Immunocytochemistry

For whole-mount immunostaining, the following antibodies were used: anti-Vas from rat (gift of P. Lasko), and monoclonal anti-GFP (JL-8) from mouse (Clontech). Immunoreactivity was detected with Alexa Fluor 488- (Molecular Probes) conjugated secondary antibodies (1∶200). Images were acquired on a Nikon Eclipse C1si laser scanning confocal microscope and processed with Adobe Photoshop and ImageJ software.
